# A recent intermezzo at the Ribosome Club

**DOI:** 10.1098/rstb.2016.0185

**Published:** 2017-03-19

**Authors:** Michael Y. Pavlov, Anders Liljas, Måns Ehrenberg

**Affiliations:** 1 Department of Cell and Molecular Biology, Uppsala University, Husargatan 3, Box 596, Uppsala 75124, Sweden; 2 Department of Biochemistry and Structural Biology, Lund University, Box 124, 22100 Lund, Sweden

**Keywords:** ribosome, translation accuracy, proofreading, initial transfer RNA selection, tautomers

## Abstract

Two sets of ribosome structures have recently led to two different interpretations of what limits the accuracy of codon translation by transfer RNAs. In this review, inspired by this intermezzo at the Ribosome Club, we briefly discuss accuracy amplification by energy driven proofreading and its implementation in genetic code translation. We further discuss general ways by which the monitoring bases of 16S rRNA may enhance the ultimate accuracy (*d*-values) and how the codon translation accuracy is reduced by the actions of Mg^2+^ ions and the presence of error inducing aminoglycoside antibiotics. We demonstrate that complete freezing-in of cognate-like tautomeric states of ribosome-bound nucleotide bases in transfer RNA or messenger RNA is not compatible with recent experiments on initial codon selection by transfer RNA in ternary complex with elongation factor Tu and GTP. From these considerations, we suggest that the sets of 30S subunit structures from the Ramakrishnan group and 70S structures from the Yusupov/Yusupova group may, after all, reflect two sides of the same coin and how the structurally based intermezzo at the Ribosome Club may be resolved simply by taking the dynamic aspects of ribosome function into account.

This article is part of the themed issue ‘Perspectives on the ribosome’.

## 1. Introduction

High resolution crystal and cryo-electron microscopy (cryo-EM) structures of the ribosome and its subunits in functional complexes have now made it possible, at least in principle, to anchor the physical chemistry of ribosome function in ribosome structure [1–4]. Such a development has been pioneered by V. Ramakrishnan who made fundamental contributions to our understanding of how the ribosome controls the accuracy of codon translation. A striking finding that came from studies of crystal structures of the 30S ribosomal subunit from *Thermus thermophilus* is that cognate and near-cognate codon–anticodon interactions lead to different structures of the decoding centre (DC), and in particular to drastically different conformations of the A1492, A1493 and G530 bases of 16S rRNA, now known as the ‘monitoring bases’ [5–7]. Additionally, in cognate cases, binding of the monitoring bases to the codon–anticodon helix leads to a domain closure of the 30S ribosomal subunit [7]. These discoveries led to a model of stereo-selective discrimination against near-cognate base pairing, where only Watson–Crick geometry of the first two positions of the codon–anticodon helix is compatible with the ‘flipped out’ conformation of the monitoring bases and their stable binding to the codon–anticodon helix. This ‘flipped out’ conformation, postulated to be a strict requirement for the GTPase activation on elongation factor Tu (EF-Tu), signals the end of initial codon selection by transfer RNA in ternary complex with EF-Tu and GTP.

Almost a decade after general acceptance of the stereo-selective model of initial codon selection, the M. Yusupov/G. Yusopova group in Strasbourg published crystal structures of the 70S ribosome from the same organism with A-site bound deacylated tRNAs with their anticodons in cognate or near-cognate interactions with mRNA codons [8–11]. They found that both cognate and near-cognate codon–anticodon helices induce nearly the same geometry of the decoding centre of the ribosome. In this, the monitoring bases are in the ‘flipped out’ conformation snugly bound to the codon–anticodon helix. This flipped out conformation is similar to that observed by the Ramakrishnan group for cognate codon–anticodon interactions in the crystal structures of the isolated 30S subunit [5,6]. These findings led Yusupov/Yusupova, E. Westhof and co-workers to propose that codon recognition occurs according to principles different from those proposed by Ramakrishnan [6]. These events and their repercussions form the ‘intermezzo’ or ‘unpleasantness’ [12] at the Ribosome Club referred to in the title of this review. Remarkably, however, we note that neither the 30S structures determined by Ramakrishnan's group nor the 70S structures determined by the Yusupov/Yusupova group have direct bearing on the actual steps during which codon selection takes place on the translating ribosome. So, from first impressions one may think that the ‘intermezzo’ is much ado about nothing. However, as we shall see, the ground-breaking structural work by Ramakrishnan, Yusupov, Yusupova and the discussions following in their wake may lead to fundamentally new insights into the physical-chemical aspects of ribosome function. This, however, requires the structural aspects of the ribosome to be positioned within the dynamics of their functional context, as recently suggested by Ramakrishnan [4,6,13]. The present text is written in this spirit of Ramakrishnan and we do hope that its themes will be further developed in the near future for a deeper integration of structure and function than is available today.

## 2. Synopsis of this work

We begin with a brief historical survey of how the ribosome tunes the accuracy of genetic code translation. Here we mention the possibility that cognate-like, rare tautomeric forms of nucleotides may contribute to the missense error frequency of the translating ribosome, but the formal aspects of such putative contributions will not be treated until the end of this work. Following the historical survey (§3), we discuss the principles of proofreading and experimental observations relevant to this accuracy amplifying mechanism. Then, we develop a kinetic model for initial codon selection by ternary complex which specifies the rate constants that are affected by Mg^2+^ concentration and aminoglycoside addition (§4). After this we discuss the accuracy predictions of the model and in particular the principles by which the monitoring bases may increase the accuracy of code translation (§5). Then, we describe in more detail the crystal structures of the 30S ribosomal subunit from the Ramakrishnan group and the 70S structures from the Yusupov/Yusupova group (§6). This is followed in §7 by a description of how aminoglycosides hyperactivate the monitoring bases and how Mg^2+^ action and aminoglycosides orthogonally corrupt the accuracy of codon reading (§8). Finally, in §9 we discuss how the existence of cognate-like rare tautomers of nucleotide bases changes the interpretation of the role of the ribosome in genetic code translation.

## 3. Brief history of ribosome contributions to the accuracy of codon translation

It is well established that the ribosome contributes to the accuracy of genetic code reading by aa-tRNAs by two different and highly efficient mechanisms [1,4,6,13].

The first mechanism, commonly referred to as proofreading, has been explained in physical terms in [14–19]. It was originally put forward to explain how the accuracy of the initial selection of a codon by aminoacyl (aa)-tRNA in ternary complex with EF-Tu and GTP can be increased by re-checking the codon–anticodon complementarity after GTP hydrolysis on EF-Tu in one [20–24] or several [25] ‘proofreading’ steps. An absolute requirement for proofreading to bypass the constraint imposed by ‘detailed balance’ [26] is that substrate discarding in proofreading must be thermodynamically driven, e.g. by hydrolysis of GTP as in codon translation [20,21,23,24], or of ATP as in aminoacylation of tRNA [27,28], at high chemical potential over their hydrolytic products [14,17,29]. In both initial selection and proofreading, there is a structurally determined ‘ultimate accuracy’ parameter, *d*, separating cognate from near-cognate substrates [16,19,30,31]. When the *d*-parameter is the same in initial selection and proofreading, one proofreading step boosts the upper accuracy limit from *d* to *d*^2^ [16,19,30]. With two proofreading steps the upper limit is *d*^3^ and so on [14,15].

By the second accuracy enhancing mechanism, the ribosome increases the structurally determined parameter *d* itself by maximizing the standard free energy difference between a cognate and a near-cognate codon–anticodon helix [32]. In concrete structural terms, we now know that this happens by activation of a small set of ‘monitoring’ bases in 16S rRNA, as indicated by Puglisi's NMR work [33,34] and clarified by Ramakrishnan and co-workers from their pioneering crystallography on the small (30S) ribosomal subunit bound to tRNA and mRNA analogues in cognate or near-cognate complexes [5,7]. The Ramakrishnan group suggested that the geometry of the monitoring bases G530, A1492 and A1493 favours their complex formation with cognate compared with near-cognate codon–anticodon helices, thereby rendering stereo-selectivity to codon recognition by tRNAs [1,5–7]. From this stereo-selection model, they predicted that cognate but not near-cognate codon–anticodon helices will display Watson–Crick geometry [1,6]. The combination of the two accuracy enhancing mechanisms, the *d*-value multiplication by proofreading and the *d*-value boosting by monitoring codon–anticodon helices, may confer extremely high accuracy to ribosome dependent genetic code translation, as now observed in living cells [35,36] and in the test-tube [20,21,37,38].

There are, however, some ‘error hotspots’ with high total error frequency in the living cell [35–37], which emanate from surprisingly high initial selection errors for, in particular, U:G mismatches in the middle codon position [38]. The existence of error hotspots appears mysterious, given the existence of accuracy amplification by both proofreading and initial selection [1,6,20,21,23–25]. It was, however, pointed out early on that mismatches may slip through all error-attenuating defence systems of the ribosome by adopting rare cognate-like tautomeric forms. In an influential paper, Topal & Fresco [39] argued that mistranslation of the genetic code to a large extent depends on tautomeric shifts of G from its keto to its enol form and of A from its amino to its imino form allowing for cognate-like base pairing of G with U and of A with C [39]. Interestingly, Topal & Fresco argued that a rare, cognate-like, tautomeric form that exists upon establishment of codon–anticodon contact will be stabilized in that protonic state all the way until peptide bond formation [39]. They also noted that this scenario would effectively preclude ribosome-dependent tuning of the accuracy of peptide bond formation and, in particular, disarm the proofreading-based error defence system of the ribosome. In this extreme form, the hypothesis of error generation primarily due to cognate-like rare tautomers is contradicted by the experimentally verified existence of proofreading and by error inducing effects of high Mg^2+^ concentration and ribosome binding drugs. However, when rapid equilibration between tautomeric forms is allowed in pertinent tRNA selection steps, then the tautomer explanation for certain error types becomes realistic and interesting. More about this is provided in §§4, 5 and 9.

## 4. Accuracy amplification by proofreading in genetic code translation

The ribosome provides the structural context for kinetic proofreading [16,19] which follows initial codon selection by aa-tRNAs as they enter the A site of the ribosome in ternary complex with EF-Tu and GTP. The proofreading mechanism is in operation after GTP hydrolysis on EF-Tu, and its substrate discard steps depend on the free energy dissipation provided by hydrolysis of GTP at high chemical potential to GDP and Pi at much lower chemical potential [17]. The chemical potential drop is determined by how much GTP is shifted above equilibrium with its hydrolytic products. This shift, which has been estimated as 10^9^-fold in the bacterial cell, provides a fundamental upper limit to the accuracy of proofreading [14], even if multiple selection steps are allowed [14,15,18]. Proofreading allows the ribosome to repeatedly use the same standard free energy difference between a near-cognate and a cognate substrate on the pathway to product formation, ΔΔ*G*^0^, which defines the maximally possible single step selectivity, *d* [16,30]
4.1
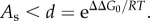


Here, *A*_s_ is the current accuracy enhancement of a single step, *R* the gas constant and *T* the absolute temperature. In each selection step, the cognate and near-cognate substrates have the probability *p*^c^ and *p*^nc^, respectively, to move forward toward product formation and the probabilities 1 − *p*^c^ and 1 − *p*^nc^, to be discarded at that step. The per step accuracy enhancement is defined as
4.2
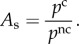


Kinetically, the situation is illustrated by two single step selection motifs
4.3
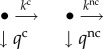


The probabilities that cognate and near-cognate substrate move to the right from state • are given by
4.4
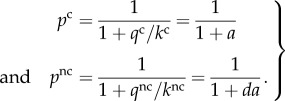


It follows directly from equations (4.2) and (4.4) that
4.5
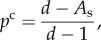
which is an elementary example of the obligatory trade-off between efficiency (*p*^c^) and current accuracy (*A*_s_) in all enzymatic selections [22,37]. Note that when the current accuracy *A*_s_ is equal to the maximal accuracy, *d*, the probability, *p*^c^, that a cognate substrate successfully passes the selection step towards product formation is equal to zero, implying a shut-down of cognate protein synthesis. Finally, we note that there are two fundamental upper limits for the overall accuracy, *A*, in genetic code translation with accuracy enhancement by proofreading. The first is related to the shift, *γ*, of the GTP concentration above equilibrium with the GDP and phosphate concentrations and the other to the number, *n*, of proofreading steps in the mechanism [14,30]
4.6
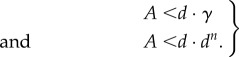


In the upper inequality, it is assumed that one GTP molecule is consumed per attempted passage through the proofreading steps. If there had been two GTP molecules per attempt, *γ* would have been replaced by *γ*^2^ and so on. The simple form of the lower inequality in equation (4.6) follows from the assumption that initial selection and subsequent proofreading steps have the same *d*-value.

In the past, it has tacitly been assumed that there is a single proofreading step in genetic code translation [23,24,37,40]. However, from recent observations the existence has been suggested of two consecutive proofreading steps (*n* = 2) in bacterial protein synthesis [25]. It has also been proposed that multiple step proofreading plays an important role in neutralizing initial selection error hotspots [37,38]. In the first proofreading step, aa-tRNA is discarded in ternary complex with EF-Tu and GDP. In the second proofreading step, after release of EF-Tu·GDP, aa-tRNA will be discarded alone, presumably from an A/T state on the ribosome [25]. In line with these proposals, cryo-EM pictures of *Homo sapiens* ribosomes from living human cells show ribosomes in complex with aa-tRNA in A/T state with (i) EF-Tu·GTP, (ii) with EF-Tu·GDP, and (iii) after EF-Tu·GDP release [41]. Taking into account that near-cognate aa-tRNA is discarded from the bacterial ribosome in complex with EF-Tu·GTP in the initial selection step, in complex with EF-Tu·GDP in the first proofreading step and alone in the second proofreading step, we propose that similar proofreading mechanisms are at work in bacteria, eukarya and, perhaps, also in archaea [25].

In the next section, we will describe a kinetic model for initial codon selection to pave the way for a functionally oriented discussion of the crystal structures of the 30S subunit [1,5–7] and the 70S ribosome behind the recent intermezzo at the Ribosome Club [8–11,42].

## 5. Accuracy of initial codon selection and its amplification by monitoring bases

A peculiar feature of the crystal structures that caused the intermezzo at the Ribosome Club is their lack of direct relevance for the process of codon selection on the mRNA translating ribosome. The structures from the Yusupov/Yusopova group picture the 70S ribosome bound to already accommodated A-site RNA with its anticodon in contact with the mRNA codon [8–11]. By contrast, in the highly dynamic situation of ‘real’ initial codon selection the tRNA structure is in a bent conformation and in complex with EF-Tu [43–45]. Concerning proofreading, experimental evidence suggests that aa-tRNAs are discarded before, rather than after, A-site accommodation [21,25,46]. We have, however, to be cautious regarding the last point, since Demeshkina *et al*. [9] report that the elbow and acceptor domains of near-cognate tRNA were highly disordered rather than properly positioned in the peptidyl transfer centre of the ribosome. This may greatly increase the dissociation propensity for near-cognate aa-tRNAs before the amino acid receives the nascent peptide chain. This could possibly lead to proofreading also of A-site accomodated aa-tRNA, as previously observed for unnatural amino acids [47].

Similarly, structures obtained by the Ramakrishnan group picture only the 30S subunit containing short stem–loop analogues rather than authentic tRNAs and the A site programmed with short oligonucleotides rather than authentic mRNAs [5,7]. This means, in other words, that there is no straightforward way to interpret the crystal structures of the Ramakrishnan [5,7] and Yusupov/Yusupova [8–11,42] groups as direct representations of actual steps in codon recognition in the dynamic context of the mRNA translating ribosome.

Bearing this in mind, we note that the striking discrepancy between the two sets of structures is that the Ramakrishnan group observed distinct 30S subunit structures for cognate codon–anticodon interactions on one hand and their near-cognate counterparts on the other hand, while the Yusupov/Yuspova group observed remarkably similar structures for, in particular, G : C cognate and G : U near-cognate base pairing in the codon–anticodon interactions. The Ramakrishnan group saw Watson–Crick pairing for cognate codon–anticodon helices supported by ordered monitoring bases and closure of the 30S subunit only for cognate and not for near-cognate codon–anticodon interactions. These structural differences were used by Ramakrishnan and co-workers to explain ribosome aided codon recognition in terms of stereo-selective checking of codon–anticodon helix geometry by ‘monitoring bases’ of the ribosome [1,5–7]. The Yusopov/Yusupova group, by contrast, observed virtually identical geometries of pre-arranged decoding centre, including activated conformation of the monitoring bases for a large set of cognate and near-cognate codon–anticodon interactions alike. Therefore, they suggested a very different type of explanation for ribosome-aided codon recognition [8–11,42]. In particular, in collaboration with E. Westhof, they suggested that the origin of errors owing to mismatches of the G : U type results from the existence of short lived tautomeric or anionic cognate-like forms of these bases that allow for the virtually perfect Watson–Crick like geometries displayed by their crystal structures. Following Topal & Fresco [39] they also suggested that upon ternary complex binding to the A site of the ribosome, a rare, cognate-like tautomer in aa-tRNA or mRNA will remain in that tautomeric form until GTP hydrolysis on EF-Tu and further until peptidyl transfer causing G : U mismatch errors in the first and second codon positions [10]. They propose, however, that A : C and many other mismatches generate errors not through tautiomerization but through the binding in a preformed decoding centre in a geometry that deviates considerably from the Watson–Crick geometry [10,42,48]. The ribosome counteracts this type of error by preventing the formation of strong H-bonds between the bases of the codon–anticodon helix which causes fast dissociation of near-cognate tRNAs from the preformed decoding centre [10,42,48].

In cases when codon translation errors are dominated by misreading of near-cognate tautomers, the assumption of blocked intramolecular proton transfer on the ribosome and thus no equilibration between tautomeric states in codon or anticodon will effectively eliminate ribosomal proofreading [39]. If, however, the tautomeric states are allowed to equilibrate during some selection steps on the ribosome, then they may have significant effects on our interpretations of the principles of error suppression by the translating ribosome. These alternative scenarios will be discussed in §9.

At the present time more is known about the structural and kinetic features of initial codon selection by ternary complex than about the proofreading reactions that occur deep into the sequential scheme leading up to the mysteries of peptidyl transfer [4,13,47,49–52]. Therefore, we will here discuss the controversial crystal structures from the contending groups of Ramakrishnan and Yusupov/Yusupova in the context of initial codon selection by ternary complex. As the starting point for this discussion, we will use a scheme with some features similar to those in previous schemes [21,40].
5.1
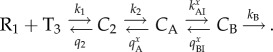


When the ribosome in post-translocation state, R_1_, associates with ternary complex, T_3_, cognate or near-cognate to the A-site codon with rate constant *k*_1_ this leads to the pre-selection complex *C*_2_ in which there is no codon–anticodon interaction [21,40]. T_3_ may dissociate from *C*_2_ with rate constant *q*_2_, or move forward to *C*_A_ (_A_ for codon–Anticodon contact) through a conformational change in aa-tRNA (tRNA bending) and concomitant establishment of codon–anticodon contact with rate constant *k*_2_. Complex *C*_A_ may move backward to *C*_2_ with rate constant 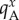, where *x* = c stands for cognate and *x* = nc for near-cognate ternary complex. Alternatively, *C*_A_ may move forward to *C*_B_ (_B_ for monitoring Bases contact) by activation of the monitoring bases with rate constant 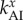 (I for Inhibitor), where I *=* 0 for no aminoglycoside in the complex and I = P for paromomycin and I = N for neomycin, etc. *C*_B_ may move backward to *C*_A_ by rate constant 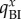 or forward by GTPase activation and GTP hydrolysis to post-GTP hydrolysis ribosomal states by rate constant *k*_B_. In scheme (5.1), four parameters (*k*_1_, *q*_2_, *k*_2_ and *k*_B_) are assumed to be insensitive and three parameters (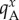, 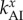 and 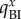) sensitive to codon–anticodon interactions. Besides, two parameters (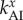 and 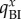) are also sensitive to the aminoglycoside status of the ribosome. Rate constants *k*_1_, *q*_2_ and *k*_2_ are codon–anticodon insensitive owing to lack of codon–anticodon contact, and the error enhancing impact of increasing Mg^2+^ ion concentration comes from a large decrease in the non-selective dissociation rate constant *q*_2_ [22,37,38]. A relatively small value of *q*_2_, rather than as previously proposed a relatively large value of the association rate constant *k*_1_ [53], accounts for the relatively large affinity of ternary complex to the ribosome at high Mg^2+^ concentration. The structurally determined selectivity of the codon–anticodon contact itself defines the *d*-value *d*_A_
5.2
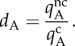


The structurally determined accuracy amplification by the monitoring bases defines the *d*-value *d*_B_
5.3
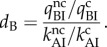


The product of *d*_A_ and *d*_B_ defines the total *d*-value for initial codon selection by ternary complex
5.4
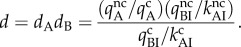


The fulcrum of initial codon selection is the difference, ΔΔ*G*_0_, between the standard free energy of GTPase activation for ternary complex with near-cognate and cognate codon–anticodon interaction. This difference is related to the *d*-value as in equation (4.1). We suggest the ‘signal’ for GTPase activation of ternary complex to be the formation of a cognate or near-cognate codon–anticodon helix in full contact with the monitoring bases, as suggested by the Åqvist group [54]. The full contact of monitoring bases was demonstrated for A-site bound tRNA in both cognate and near-cognate contexts [8,9,11,42]. We also assume here that the GTPase activation rate constant *k*_B_ is similar in cognate and near-cognate cases. For the G : U mismatches in the first two positions of the codon this assumption is well justified by the identical geometry of near-cognate and cognate codon–anticodon helices observed in all such cases [9,11]. In the cases of other mismatches, such as A : C and U : U, for example, this assumption is a bit tentative since the geometry of the codon–anticodon helix with these mismatches deviates from that of the cognate one despite the identical arrangement of the monitoring bases in the ribosome decoding centre [42,48]. The rate constant may, in such cases, be smaller in near-cognate than in cognate cases as suggested [21,40].

## 6. Controversial crystal structures in functional context

While Yusupov/Yusupova have observed ribosomes with deacylated tRNA in the A site equilibrated over very long times in rigid crystals, scheme (5.1) suggests a radically different, dynamic picture. We know from ternary complex titrations at different temperatures that the time for a ribosome to transcend through its *C*_2_, *C*_A_ and *C*_B_ complexes to GTP hydrolysis is about 1 ms at 37°C under the *in vivo* compatible conditions of our biochemistry [30]. This implies that the forward rate constants in scheme (5.1) for transitions between *C*_2_, *C*_A_ and *C*_B_ complexes must all be larger than 1000 s^−1^, meaning that aa-tRNA in ternary complex will shift from its canonical to its bent conformation for codon–anticodon interaction, stimulate monitoring base activation and adopt a conformation which allows for rapid GTP hydrolysis within a time span below the resolution of stopped-flow, quench-flow and virtually all single molecule spectroscopy designs. From the properties of scheme (5.1), we expect that, in experiments with non-hydrolysable analogues of GTP or with GTPase deficient mutants of EF-Tu, near-cognate ternary complexes will preferentially be in state *C*_2_, cognate ternary complex preferentially in state *C*_B_ and both types of ternary complexes may be transiently present in state *C*_A_. In these respects, scheme (5.1) of this work is distinct from the scheme of initial selection proposed by the Yusupov/Yusupova group on the basis of their crystal structures [9]. Their scheme does not contain state *C*_A_ of scheme (5.1), and suggests the existence of a preformed decoding centre with activated monitoring bases acquiring their final *C*_B_ like-conformation already in state *C*_2_, well before the entry of the tRNA anticodon in the decoding centre [9]. We propose therefore that state *C*_B_ in scheme (5.1) ‘corresponds’ to their tRNA bound state, although one may bear in mind that aa-tRNA is EF-Tu-bound and bent in *C*_B_, and furthermore, that *C*_B_ does not originate directly in state *C*_2_. Instead, it originates in state *C*_A_ which becomes *C*_B_ upon monitoring-base activation. Concerning the absence of state *C*_A_ in their crystal structures, it may be ascribed to extreme conditions of crystallization with respect to buffer, temperature and excluded volume conditions which favour state *C*_B_ in relation to *C*_A_ not only for cognate but also for near-cognate tRNAs [13,55].

We suggest that in the normally short-lived state *C*_A_ of scheme (5.1) the monitoring bases are largely disordered or bound within the 16S rRNA, the cognate codon–anticodon helices have Watson–Crick geometry but their near-cognate counterparts do not and may instead acquire a wobble geometry at any of the three codon positions. The Ramakrishnan structures of the 30S subunit in complex with near-cognate tRNA acceptor stems may correspond to state *C*_A_ which is much less ordered than state *C*_B_ and has a negligible rate of GTP hydrolysis. This would mean that both the 30S subunit complexes of Ramakrishnan and the 70S ribosome complexes of Yusupov/Yusupova are relevant to the initial codon selection process when placed in their correct functional context. In line with one of our early proposals [32], we also suggest that the highly ordered codon–anticodon conformation in state *C*_B_ maximizes the difference in standard free energy between cognate and near-cognate codon–anticodon helices. The implication here is that the conformational state of the codon–anticodon interaction that promotes GTP hydrolysis in ternary complex is also the conformational state that maximizes the standard free energy difference between right and wrong substrates.

How, then, can the monitoring bases increase the accuracy of initial codon selection by ternary complex? At this point we discern two major options. The first concerns bringing the comparatively disordered state *C*_A_ to the highly ordered state *C*_B_ for GTPase activation in scheme (5.1), which in near-cognate cases corresponds to a large standard free energy increase. In cognate cases, by contrast, the codon–anticodon helix is similar in states *C*_A_ and *C*_B_ so the standard free energy increase in the *C*_A_ to *C*_B_ transition is expected to be negligible, as suggested [1,4,6,7]. This type of accuracy amplification is accounted for by the selection parameter *d*_B_ defined in equation (5.3) above. The other option is that activation of the monitoring bases creates a water free environment for deciphering the two first bases of the codons of mRNAs [54]. In near-cognate cases, the standard free energy increase by missing hydrogen bonds for non-matching bases may, in a water proficient environment, be reduced by hydrogen bonds between bases and water molecules. In a water deficient environment, by contrast, this compensation is not possible which would make the standard free energy difference between a missing and an existing base-to-base hydrogen bond much larger than in the water proficient case [54]. Also this amplification would be accounted for by the factor *d*_B_ in equation (5.3) above.

## 7. Mechanism of aminoglycoside corruption of the accuracy of initial codon reading

It has been known for a long time that binding of aminoglycoside drugs to the mRNA translating ribosome activates the monitoring bases A1492 and A1493 by displacing them from their ‘flipped in’ conformation inside helix h44 of the 16S rRNA and possibly also by restricting the conformational space of their disordered ‘flipped out’ state [5–10,33,34]. This suggests that aminoglycosides increase the rate constant for monitoring base activation, 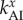 (I refers to Inhibitor, aminoglycoside) and perhaps also decrease the rate constant for monitoring base inactivation, 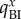 in scheme (5.1), thereby decreasing the equilibrium constant 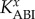 that connects states *C*_A_ and *C*_B_
7.1
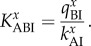


It should be clear by now that this explanation for aminoglycoside action based on large shifts of the equilibrium towards *C*_B_ state in scheme (5.1) is not compatible with the notion of a pre-formed decoding centre with the monitoring bases already flipped out to their final positions in the absence of codon–anticodon interaction in the A site of the ribosome [8–10]. This notion corresponds formally to the case of scheme (5.1) in which the equilibrium constant 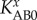 is much smaller than one for both cognate (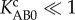) and near-cognate (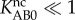) ternary complex so that the accuracy would be insensitive to their further reduction by aminoglycoside addition.

We note that the decrease of the equilibrium constant 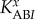 by aminoglycoside addition may well be sensitive to what particular type of aminoglycoside that is ribosome bound. A next question is whether the aminoglycoside-induced shift in the equilibrium constant 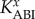 depends on whether the codon–anticodon interaction is cognate or near-cognate, i.e. whether the following equality holds:
7.2
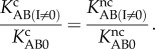


The answer to whether equation (7.2) holds may be found in the ribosome structures that show cognate and near-cognate ternary complex in a ribosomal state with activated monitoring bases and by hypothesis similar to *C*_B_ in the absence and presence of aminoglycosides [8–10]. The codon–anticodon geometries and the overall arrangements of the decoding centre were found to be remarkably similar with and without aminoglycosides, and on the basis of this information, we propose that aminoglycosides induce equal decrease in the cognate, 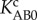, and near-cognate, 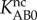, equilibrium constants so that equation (7.2) is valid. How, then, can an aminoglycoside induced shift, which is the same for the equilibrium constants 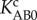 and 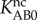, result in the large error increase observed in response to the presence of aminoglycosides on the translating ribosome *in vivo* and *in vitro* [56]? Superficially, such a mechanism affects cognate and near-cognate ternary complex alike and, yet, induces a large increase in the error of initial codon selection. To illustrate this mechanism, we simplify scheme (5.1) one step further for a closer look at the tuning of the accuracy of initial codon selection by both Mg^2+^ ions and aminoglycosides.

## 8. Tuning the accuracy of initial codon selection by aminoglycosides and Mg^2+^ at unaltered *d*-values

If we allow rapid equilibration of ribosomal states *C*_A_ and *C*_B_ compared with other steps of scheme (5.1), it can be contracted to the three step scheme
8.1
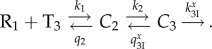


Here, the compounded rate constants 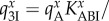 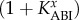 and 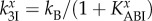 are derived by elimination of fast variables [57]. Defining 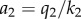 and 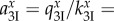 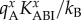 the *k*_cat_/*K_m_* values for cognate (*x* = c) and near-cognate (*x* = nc), GTP-hydrolysis on EF-Tu are given by
8.2
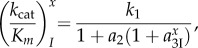
where the ultimate, intrinsic discrimination parameter *d* defined in equation (5.4) for a particular pair of cognate and near-cognate codon–anticodon interactions is expressed as
8.3
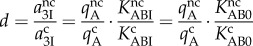


The last equality in equation (8.3) comes from the assumption of equation (7.2) that aminoglycoside binding induced the same shift in the equilibrium constants 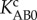 and 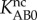 (see §7). Equation (8.3) implies therefore that aminoglycoside addition decreases 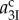 and 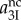 by the same, aminoglycoside specific factor, so that the intrinsic discrimination parameter, *d*, remains unaltered. According to equation (8.2), the accuracy of initial codon selection is given by
8.4
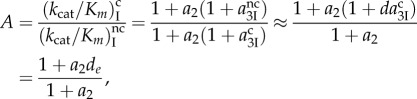
where the approximation in the second to last expression of equation (8.4) is valid by the assumption that 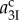 is much smaller than unity. We now propose that increasing Mg^2+^ ion concentration decreases *a*_2_ without any other effect on the accuracy. It is seen then that when the non-discriminating discard parameter *a*_2_ increases at decreasing Mg^2+^ concentration from values much smaller than unity to values much larger than unity, the accuracy increases from its lowest value *A*_min_ = 1 toward its highest asymptote *d_e_*
8.5
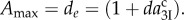


When A is changed by variation of the parameter *a*_2_ with no change in *d_e_*, a plot of the efficiency of cognate codon reading, (*k*_cat_*/K_m_*)^c^ versus A gives a straight line [22,37,38]
8.6
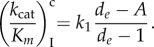


We note here that the titration with Mg^2+^ ions leads to an asymptotic limit considerably smaller than the intrinsic selectivity parameter *d*. The reason is that Mg^2+^ ion titration only affects the parameter *q*_2_ and thus leaves the rate constants connecting states *C*_A_ and *C*_B_ unchanged. This implies that in cognate, but not near-cognate cases, *C*_B_ is much below equilibrium with *C*_A_ corresponding to 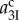-values considerably smaller than unity. It is also seen that when addition of aminoglycosides reduces the non-discriminating discard parameter 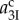 the accuracy decreases in proportion to the 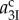 decrease as long as 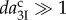 and 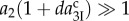. We suggest, in summary, that Mg^2+^ ions and aminoglycosides corrupt the accuracy of initial codon selection orthogonally: increasing Mg^2+^ concentration decreases the non-discriminating parameter *a*_2_ and aminoglycoside addition decreases 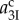.

It was previously observed that the maximal rate of GTP hydrolysis is much smaller for near-cognate than for cognate ternary complex reactions [21,40] and that this maximal rate increases greatly with aminoglycoside addition [56]. From these maximal rate data, it was suggested that the rate constant, *k*_B_, for GTPase activation is smaller for near-cognate than for cognate ternary complex and that both [Mg^2+^] increase [56] and aminoglycoside addition increase *k*_B_ in near-cognate but not in cognate cases by a mechanism referred to as ‘induced fit’ [40,46]. A fundamental difference between these previous proposals and the present one is that the former imply decreasing *d*-values in response to increasing Mg^2+^ concentration and aminoglycoside addition, while the *d-*values are unchanged in the present model. This distinction is essential, because it decides how to interpret high resolution structures of the ribosome in the presence and absence of aminoglycosides [5–10]. As discussed above, this is related to how cognate codon–anticodon interactions in the decoding centre of the ribosome signal rapid GTP hydrolysis 70 Å away in the GTPase centre of EF-Tu. If, as suggested by Satpati *et al*. [54], the ‘signal’ is simply the positioning of a rigid ternary complex for optimal contact of its GTPase centre with the bases of 23S rRNA, then it follows that similar codon–anticodon geometries observed for cognate and near-cognate ternary complex in the presence and absence of aminoglycosides should result in similar *k*_B_-values.

Experimental proofs that the *k*_B_-value is much smaller for near-cognate than cognate ternary complex but becomes equal in the two cases in the presence of aminoglycosides were in the past provided by estimates of the maximal rate of GTP hydrolysis [56]. That the maximal rate of GTP hydrolysis (*k*_cat_) is smaller for near-cognate than cognate ternary complex and that the near-cognate but not the cognate rate increases with aminoglycoside addition are also predicted by our model of aminoglycoside action. That is, the inverse of the maximal rate, equal to the minimal time leading up to GTP hydrolysis in *T*_3_ for cognate (*x* = c) and near-cognate (*x* = nc) codons, is given for scheme (8.1) by
8.7
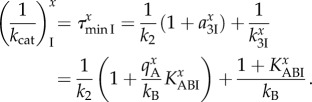


As, by hypothesis, 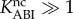 while 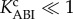 and 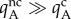 it follows from equation (8.7) that *k*_cat_ is much smaller in near-cognate than in cognate cases and increases with decreasing 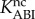 upon aminoglycoside addition. This means, in other words, that although the forward rate constant *k*_B_ in equation (8.7) may be the same for cognate and near-cognate codon–anticodon interactions and independent of the presence of aminoglycosides, the maximal rate of the GTP hydrolysis reaction in equation (8.7) is sensitive both to the type of codon–anticodon match and presence or absence of aminoglycosides, as previously observed experimentally but interpreted differently [56]. It also follows that the maximal rate of the cognate reaction is virtually independent of the presence or absence of aminoglycosides, as observed experimentally [56], and can be approximated as
8.8
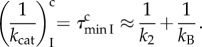


## 9. Do rare tautomeric base-forms limit the accuracy of code translation?

From the striking observations by recent X-ray crystallography of virtually identical conformations of cognate and near-cognate codon–anticodon complexes containing G : U mismatches [8–10], it has been suggested that rare tautomeric forms of nucleotide bases may be the primary cause for misreading of G : U base pairs in first and second codon positions [10] as originally suggested by Topal & Fresco [39]. The rationale here is that even if rare tautomeric states have high standard free energies [55], they will allow for the perfect Watson–Crick geometries observed by the Yusupov/Yusupova group and, by inference, explain the comparatively high error frequencies associated with, in particular, G : U mismatches in the first two codon positions as observed *in vivo* [35,36] and *in vitro* [22,37,38]. Following Topal & Fresco, it was considered that intramolecular proton exchange could be slow for nucleotide bases in the codons of ribosome bound mRNAs and the anticodons of ribosome bound tRNAs so that rare tautomeric states may remain frozen in their cognate-like conformation until the selection process is over [48]. However, another extreme alternative, i.e. that tautomeric configurations remain in rapid equilibrium throughout the selection process, is made more likely by NMR data on rare tautomeric states in RNA helices [58]. Here, we will inspect three extremes regarding the intramolecular rates of conversion between tautomeric forms of nucleotide bases in ribosome bound ternary complex for confrontation with experimental data on initial codon selection. To make the argument concrete we will assume that a nucleotide base in ternary complex bound tRNA is involved in a near-cognate interaction with first or second base in the codon triplet. The effective intrinsic discrimination against the major tautomeric form of the base is *d_e_* (see equation (8.5)) and in the absence of a rare tautomer the accuracy of initial codon selection would be given by equations (8.4) and (8.5) above. A rare tautomeric form, which appears as a cognate base in the ternary complex selection, occurs with frequency 1/(*K* + 1) in its free state, whereas the abundant tautomer occurs with frequency *K*/(*K* + 1), where 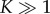. First, we assume that as soon as a ternary complex is ribosome bound in state *C*_2_ of scheme (5.1) there is no intramolecular proton exchange so that both tautomeric states are frozen in the states they had upon ternary complex binding to the A/P state of the ribosome. Second, we assume that there is rapid exchange between tautomeric states in *C*_2_, where there is no codon–anticodon contact but not in subsequent complexes. Third, we assume rapid exchange of tautomeric states all the way up to peptidyl transfer. Elementary considerations show that in the first case the accuracy is given by (compare with equation (8.4) above)
9.1
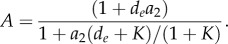


**Figure 1.**
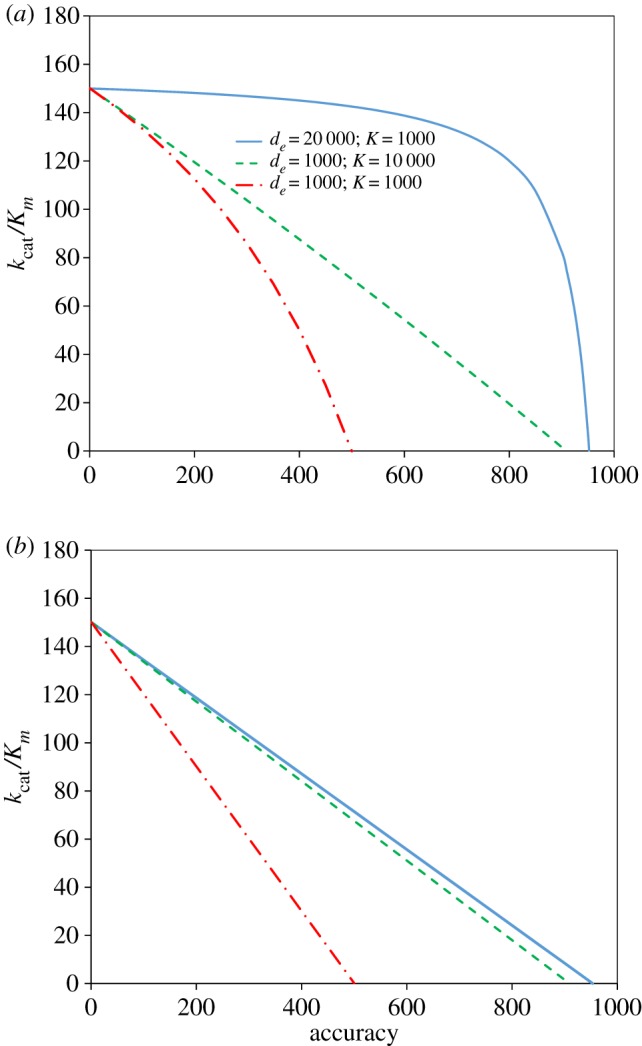
Illustrative efficiency–accuracy trade-off plots with error induction by cognate-like, rare base tautomers in the anticodon of ternary complex (T_3_) for initial codon selection according to scheme (5.1). The cognate codon reading efficiencies, *k*_cat_*/K_m_*, are plotted as functions of the accuracy, defined as the ratio between cognate and near-cognate *k*_cat_*/K_m_*-values. Ultimate discrimination against the abundant tautomer is *d_e_* and the equilibrium constant between abundant and rare tautomer in free T_3_ is *K*. Blue solid lines for *d_e_* = 20 000, *K* = 1000, green dashed lines for *d_e_* = 1000, *K* = 10 000 and red dash-dot lines for *d*_*e*_=1000, *K*=1000. (*a*) Rapid equilibration between rare and abundant tautomers of T_3_ in free and slow equilibration in all ribosome bound forms of T_3_, as in equation (9.1). (*b*) Rapid equilibration between rare and abundant tautomers of T_3_ in free and *C*_2_-complex bound forms and slow equilibration in all other ribosome bound forms of T_3_ as in equation (9.2).

In the limit of very high *K*-value the accuracy *A* in equation (9.1) is equal to *A* in equation (8.4). In the limit of very high discrimination, *d_e_*, against the abundant tautomer, then *A* = *K* independently of the value of the *a*_2_ parameter, so that in this limit the accuracy becomes independent of the efficiency of the cognate reaction. It is clear, therefore, that in plots of the efficiency of cognate codon reading versus accuracy as in equation (8.6), there will in general be no straight lines, in contrast with what has been observed experimentally [22,37,38]. This is illustrated in figure 1*a*.

In the case of rapid equilibration of tautomeric states in state *C*_2_ but not in subsequent states with established codon–anticodon contacts the accuracy is given by
9.2
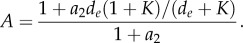


When *K* is much larger than *d_e_*, the accuracy is determined by *d_e_*, and when *K* is much smaller than *d_e_* the error is dominated by *K*. In this case, in contrast with the previous one, plots of the efficiency of cognate codon reading versus accuracy as in equation (8.6) will always result in straight lines. This is illustrated in figure 1*b*. However, if the tautomeric states remain frozen all the way to peptide bond formation, subsequent proofreading selection will not contribute to the accuracy [39], because the tRNA will remain in its cognate-like state and pass through the proofreading steps to peptidyl transfer with the same probability as a cognate aa-tRNA.

In the case of rapid tautomer equilibration throughout all steps leading to peptidyl transfer, proofreading can play an important role in the reduction of the error level, as observed experimentally [22,37,38]. Because we observe perfect straight lines for the efficiency–accuracy trade-off in initial selection of a large set of ternary complex misreading their near-cognate codons, and a considerable contribution of the proofreading in total error reduction, the tautomeric forms must equilibrate rapidly on the time-scale of initial selection under our experimental conditions, i.e. in the sub-millisecond range [22,37,38]. It follows from these considerations that rare tautomeric forms of nucleotide bases may be compatible not only with existing structural and but also biochemical data. The strict conditions for this are that their intramolecular conversion rates are sufficiently high to allow for the straight lines that characterize the efficiency–accuracy trade-off of initial codon selection [22,37] and for the great accuracy enhancement by proofreading [38,40,59]. We expect that future experiments with improved structural and kinetic resolution will provide an answer to the question of whether rare base tautomers are responsible for the recently [22,35–38] and previously [60,61] discovered error hotspots in bacterial mRNA translation.

## 10. Conclusion

Ramakrishnan and co-workers used a set of crystal structures of the 30S ribosomal subunit and Yusupov/Yusupova and co-workers used a set of crystal structures of the 70S ribosome to uncover the physical-chemical principles of the accuracy of genetic code translation by ribosomes. From the two sets of structures, the two groups arrived at apparently contradictory conclusions regarding the accuracy of genetic code reading, the accuracy amplifying effects of the ‘monitoring’ bases of 16S ribosomal RNA and the accuracy corruption by the presence of aminoglycoside antibiotics. These contradictions caused the intermezzo at the Ribosome Club.

Here, we have tried to relate the crystal structures of the 30S ribosomal subunit from the group of Ramakrishnan and the 70S ribosome from the group of Yusupov/Yusupova to the functional context of initial codon selection. This includes the binding of aa-tRNA in ternary complex with EF-Tu and GTP to the ribosome which involves a state without codon–anticodon contact (*C*_2_), a ternary complex bound state with codon–anticodon contact (*C*_A_) and a highly ordered state with codon–anticodon contact and the monitoring 16S ribosomal RNA bases bound to the codon–anticodon helix (*C*_B_). During initial codon selection the ribosome moves through all these states to GTP hydrolysis on EF-Tu in less than a millisecond. We draw the following conclusions.

Neither of the two sets of crystal structures has a direct bearing on the initial codon selection steps of the mRNA translating ribosome or account for the binding of ternary complex into state *C*_2_. Both sets of structures picture a very similar arrangement of the decoding centre for cognate codon–anticodon interactions that may correspond to those in the transient state C_B_ in which the monitoring bases are in their final, activated positions.

In the Yusopov/Yusupova crystal structures of the ribosome, both cognate and near-cognate codon–anticodon helix interact in almost identical manner with activated monitoring bases in a pre-formed decoding centre. These complexes may in a functional context all correspond to the highly transient *C*_B_ complex, rapidly formed not by tRNA entering a pre-formed decoding centre but by a chain of events leading from free ternary complex to *C*_2_ to *C*_A_ to *C*_B_. Aminoglycoside binding has little effect on the codon–anticodon helix conformation and the positioning of the monitoring bases in these C_B_ structures. Their model with a preformed decoding centre does not explain error induction by aminoglycosides in a compelling manner.

In the Ramakrishnan crystal structures of the 30S ribosomal subunit, near-cognate tRNAs display codon–anticodon helix conformations deviating from the Watson–Crick geometries of the cognate cases and the monitoring bases are partially inactive and disordered. These complexes may in a functional context correspond to the highly transient *C*_A_ complexes. Aminoglycoside binding has large effects on the activation of the monitoring bases and their binding to the codon–anticodon helix of near-cognate tRNAs. In a functional context these effects of aminoglycosides may correspond to their ability to induce a large shift in the equilibrium constant between *C*_A_ and *C*_B_ which in near-cognate cases increases the population of *C*_B_ and thereby degrades the accuracy.

Positioning of the crystal structures from Ramakrishnan and Yusupov/Yusopov in functional contexts suggests that they represent different sides of the same functional coin and that deeper understanding of ribosome function will require continued iterations of new structures positioned in ever better described functional contexts. In this way, the current unpleasantness at the Ribosome Club may be resolved by the benefits of its aftermath.
